# Role of epithelial-mesenchymal markers in predicting metastasis of papillary thyroid carcinoma: A retrospective case-control study

**DOI:** 10.5339/qmj.2025.45

**Published:** 2025-06-30

**Authors:** Iqbal Amer, Esraa Aldujaily, Ali Alfatlawi

**Affiliations:** 1 College of Dentistry Medicine, Jabir ibn Hayyan Medical University, Kufa, Iraq; 2 College of Medicine, Department of Pathology and Forensic Medicine, University of Kufa, Kufa, Iraq; 3 Information Technology Research and Development Center, University of Kufa, Kufa, Iraq *Email: esraa.aldujaily@uokufa.edu.iq

**Keywords:** Papillary thyroid carcinoma, epithelial-mesenchymal transition, E-cadherin, zinc finger E-box-binding homeobox 1, lymph node metastases

## Abstract

**Background:**

Papillary thyroid carcinoma (PTC) accounts for 90% of thyroid malignancies, with lymph node metastasis being a critical prognostic factor. However, the mechanisms driving metastasis remain unclear. This study investigated the role of E-cadherin and zinc finger E-box-binding homeobox 1 in cancer progression among Iraqi PTC patients.

**Methods:**

The expression levels of E-cadherin and zinc finger E-box-binding homeobox 1 were analyzed in 50 Iraqi patients diagnosed with PTC without lymph node metastases, evaluated between January 2015 and December 2021. The Pearson correlation coefficient between these markers and the risk of lymph node metastasis, as well as their relationship to tumor grade and stage, was calculated. The area under the receiver operating characteristic (ROC) curve for both markers was performed. The Medical Ethics Committee of the Iraqi Ministry of Higher Education and Scientific Research (Reference No. 14) approved the study.

**Results:**

This study examined 50 PTC cases, revealing significant associations between E-cadherin and zinc finger E-box-binding homeobox 1 expression and lymph node metastasis, clinical stage, and tumor grade. Zinc finger E-box-binding homeobox 1 expression was higher in metastatic cases (92% vs. 16%), while E-cadherin was lower (24% vs. 84%). Zinc finger E-box-binding homeobox 1 positively correlated with lymph node metastasis (*r* = 0.68), stage (*r* = 0.72), and grade (*r* = 0.56), whereas E-cadherin showed negative correlations (*r* = −0.60, −0.59, and −0.52, respectively). ROC analysis showed areas under the curve of 0.88 zinc finger E-box-binding homeobox 1 and 0.82 (E-cadherin), suggesting their potential as biomarkers for metastasis prediction.

**Conclusions:**

The study concluded that E-cadherin and zinc finger E-box-binding homeobox 1 are key predictors of lymph node metastasis, tumor grade, and stage in PTC. Monitoring these markers could enhance clinical decision-making and patient management.

## INTRODUCTION

Papillary thyroid carcinoma (PTC) is the most common type of thyroid cancer, accounting for approximately 90% of all thyroid malignancies. Its incidence has risen significantly over the past four decades, driven by advancements in diagnostic techniques such as high-resolution ultrasound and fine-needle aspiration biopsy. Experts project that by 2030, thyroid cancer, particularly PTC, will rank as the fourth most common cancer globally.^1^ Despite its excellent prognosis and low mortality rates, over 25% of PTC patients experience recurrence, highlighting challenges in management.^2–4^

Pathologically, PTC is characterized by unique nuclear features, including nuclear grooves, pseudoinclusions, and overlapping chromatin. Variants such as the follicular variant can complicate diagnosis due to overlapping histological features with benign thyroid lesions. Immunohistochemistry has emerged as a critical tool in differentiating PTC from other thyroid conditions, utilizing markers such as E-cadherin and zinc finger E-box-binding homeobox 1 (ZEB1). These markers are pivotal in understanding epithelial-mesenchymal transition (EMT), a process central to metastasis and cancer progression.^5^

EMT involves the loss of epithelial markers and cell adhesion, promoting invasive and metastatic behavior in PTC. This biological process underscores the need for molecular markers to refine diagnostic accuracy and guide treatment. Misdiagnosis due to overlapping histological features and interobserver variability among pathologists can lead to inappropriate treatments, underscoring the importance of robust immunohistochemical and molecular approaches. Treatment options for PTC typically include surgery, radioactive iodine therapy, and thyroid-stimulating hormone suppression therapy. While these methods yield high survival rates, recurrence remains a significant concern, necessitating ongoing monitoring and tailored therapeutic strategies.^5–7^

This study aims to investigate the expression of E-cadherin and ZEB1 as critical markers in PTC, particularly their roles in lymph node metastasis, tumor progression, and EMT. By elucidating their clinical significance, the research seeks to enhance diagnostic precision and inform targeted therapeutic interventions for better patient outcomes.^6,7^

## METHODOLOGY

This retrospective case-control study examined PTC patients from the Middle Euphrates region of Iraq, utilizing clinical data collected at the Middle Euphrates Oncology Hospital in Najaf, a tertiary cancer referral center, between January 2015 and December 2021. Clinical records of 50 PTC patients were reviewed, including data on gender, age, lymph node metastasis, tumor grade, stage, and histological type. Diagnostic confirmation was achieved using biopsy, computed tomography, cytology, histology, and magnetic resonance imaging. Paraffin-embedded tissue blocks were obtained from the pathology department of the main teaching hospital in Najaf for analysis.

Initially, the dataset comprised 99 cases, categorized into two groups: 59 with lymph node metastases (LNM) and 40 without. To ensure balanced representation, 25 cases were randomly selected from each group for final analysis. This approach enabled a robust comparison of clinical and pathological features associated with lymph node metastasis. Inclusion Criteria: All cases of PTC diagnosed were between January 2015 and December 2021. Complete clinicopathological data is available for each patient. Exclusion Criteria: Patients with mental deficits that prevented proper follow-up and documentation. Patients with incomplete clinicopathological records. The reason for selecting specific cases and controls was to ensure a balanced and reasonable comparison between patients with and those without LNM. Immunohistochemical staining was performed on formalin-fixed paraffin-embedded sections of PTC using the Sunlong Biotech Co. IHC kit, following the manufacturer’s instructions. The kit included hydrogen peroxide, DAB solutions, hematoxylin, and a rabbit enzyme-labeled secondary antibody. Citrate buffer (pH 6.0) and PBS buffer (pH 7.2–7.4) were used. Anti-E-cadherin (rabbit monoclonal, LSBio, Cat# LS-C92224-100) and anti-ZEB1 (rabbit polyclonal, LSBio, Cat# LS B15061) primary antibodies were employed to detect E-cadherin and ZEB1 expression, respectively. The ZEB1 antibody was diluted 100-fold, while the E-cadherin antibody was used undiluted.

### E-cadherin IHC interpretation

E-cadherin was evaluated, and its values were as follows:^8^

### ZEB1 IHC interpretation

ZEB1 was evaluated, and its intensity was categorized as follows:^9^

The study was conducted in accordance with the Declaration of Helsinki (as revised in 2013). The Medical Ethics Committee of the Iraqi Ministry of Higher Education and Scientific Research (Reference No. 14) also approved the study, waiving individual consent for this retrospective case-control study. This study used the Pearson correlation coefficient (*r*) and the *p* value to investigate the relationships between the levels of E-cadherin and ZEB1 and clinical and pathological factors, such as lymph node metastasis, tumor grade, and clinical stage. The distribution of patient demographics, tumor characteristics, and biomarker expression levels across different subgroups was described using percentage (%) calculations.

## RESULTS

Data were analyzed from 50 patients with PTC. Most were under 50 years old. The mean age was 38 years, and the range was 18–92 years, as shown in Table 1. Regarding the tumor grade, 18 patients (36%) and 32 patients (64%) received grades I and II, respectively. Regarding the clinical stages of the patient’s tumor, stage I included 4 (8%), stage II included 3 (6%), stage III included 20 (40%), stage IV 5 (10%), and 18 (36%) included unknown clinical stage. LNM were present in 25 (50%) of reported cases and absent in 25 (50%), as illustrated in Table 1. Half of the patients were cases (with LNM), while the other half were controls (without LNM).

**Figure 1:**
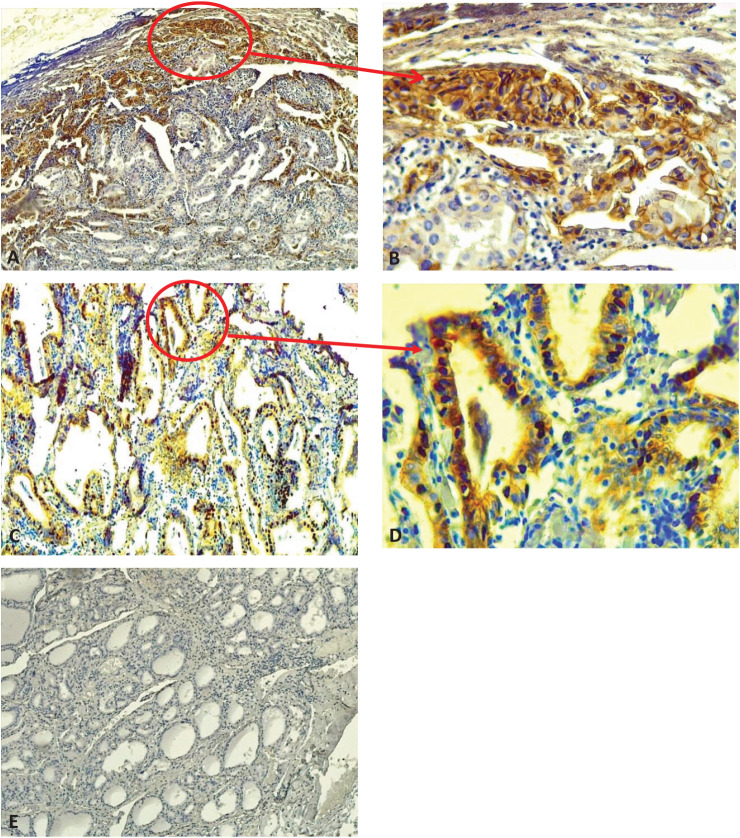
E-cadherin and ZEB1 immunohistochemical expression. (A) Strong membranous brown staining for E-cadherin (10×). (B) Strong membranous brown staining for E-cadherin (40×). (C) Strong nuclear brown staining for ZEB1 (10×). (D) Strong nuclear brown staining for ZEB1 (40×). (E) Negative control (10×) with omitted primary antibodies. Arrows indicate marker positivity in tumor cells. Magnification noted.

**Figure 2:**
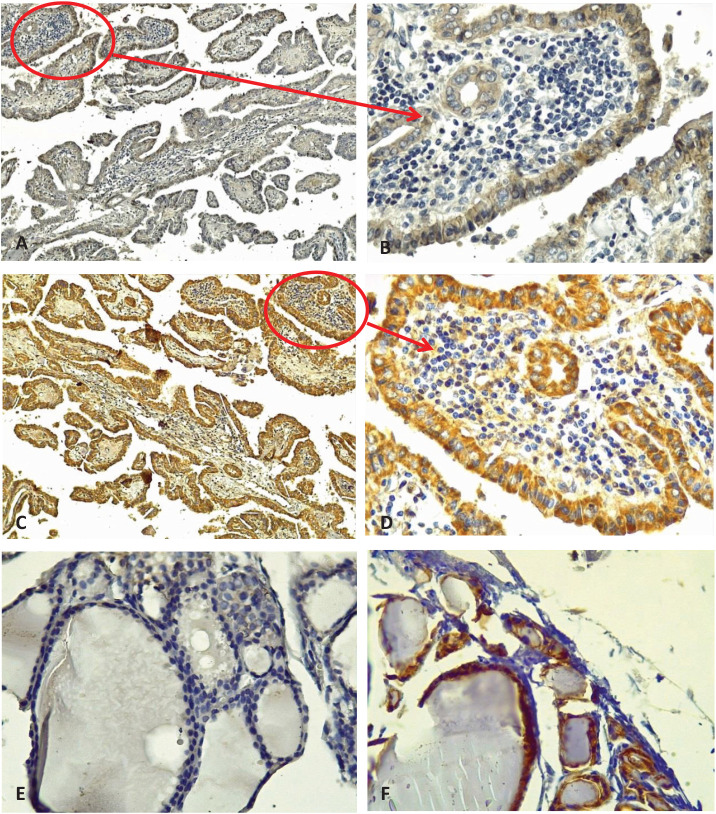
Reciprocal expression of E-cadherin and ZEB1 in normal and cancerous thyroid tissue. (A) Negative E-cadherin expression in PTC (10×). (B) Negative E-cadherin expression in PTC (40×). (C) Positive ZEB1 expression in PTC (10×). (D) Positive ZEB1 expression in PTC (40×). (E) Negative ZEB1 staining in normal thyroid tissue (40×). (F) Positive E-cadherin staining in normal thyroid tissue (40×). Arrows indicate marker positivity. Magnification noted.

**Figure 3:**
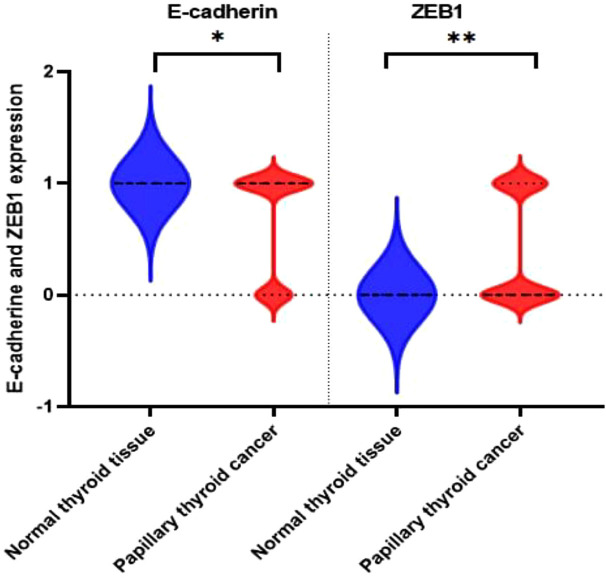
Violin plot showing immunohistochemical expression of ZEB1 and E-cadherin in malignant and normal thyroid tissues. Sections are classified as negative (0) and positive (1), with red indicating positive expression and blue indicating negative expression.

Table 2 shows the demographic data of the 50 samples in terms of E-cadherin and ZEB1 expression. ZEB1 expression was positive in 23 (92%) cases and 4 (16%) control patients and negative in 2 (8%) cases and 21 (84%) control patients as shown in Table 2. As shown in Table 2, E-cadherin expression was positive in 19 (76%) cases and negative in 4 (16%) control patients. It was negative in 6 (24%) cases and 21 (84%) control patients. We examined the expression of E-cadherin in malignant PTC and adjacent normal tissue sections. Each experiment was performed using both positive control slides and negative control slides, and the primary antibody omitted to ensure quality control and performance of the primary antibodies (Figure 1). In addition, whenever available, the expression of E-cadherin and ZEB1 was assessed in adjacent normal tissue (Figure 2). When examining the H&E slides under the light microscope, normal thyroid tissue sections were found adjacent to the malignant tissue sections in 22 of the 50 PTC cases. In our immunohistochemical analysis, 22 of 50 (44%) sections of normal thyroid tissue showed positive membranous immunostaining for E-cadherin expression, which was significantly different from cancerous tissue. None showed positive staining for ZEB1 with a significant difference compared to malignant thyroid tissue (Figure 3).

**Figure 4:**
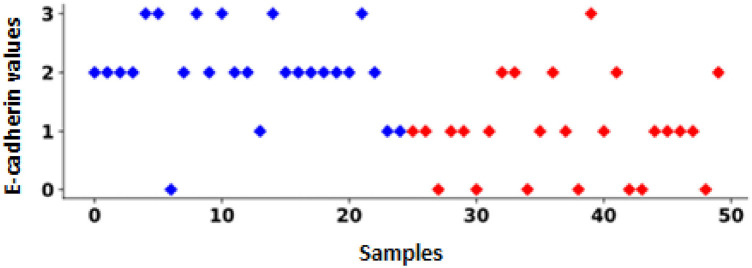
Relationship between E-cadherin expression and LNM. This figure displays E-cadherin values for all 50 cases in the immunohistochemical analysis. Colors indicate LNM status, with red representing positive cases and blue representing negative cases. Four cases were negative for LNM.

For statistical analysis, the Pearson correlation coefficient (*r*) was used to evaluate the relationship between expression level and clinicopathological parameters. Table 3 shows the Pearson correlation coefficient (*r*) and *p* value for the 50 PTC cases examined using immunohistochemistry to check the expression of E-cadherin and ZEB1. Interestingly, a significant negative Pearson correlation was found between E-cadherin and LNM. With a Pearson correlation coefficient of −0.60 (*p* = 3.76 × 10^−6^), E-cadherin immunostaining was found in 21/25 (84%) node-negative PTC, while only 6/25 (24%) node-negative PTC patients had E-cadherin immunostaining. In 50 cases of PTC, the scatter plot shows the correlation between E-cadherin expression and LNM. Red dots indicate LNM-positive cases, while blue dots indicate LNM-negative cases. In general, blue dots are associated with higher E-cadherin levels. This inverse relationship supports the potential role of E-cadherin as a biomarker to assess the risk of metastasis in PTC, as higher levels are associated with a lower risk of lymph node metastasis, while lower levels are more commonly observed in metastatic cases (Figure 4).

**Figure 5:**
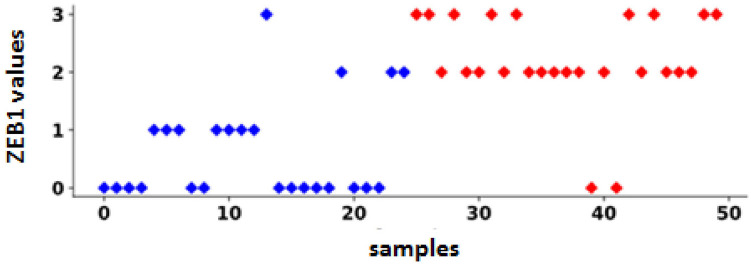
The relationship between ZEB1 expression and lymph node metastasis (LNM). This figure shows ZEB1 values for all 50 cases in the immunohistochemical analysis. Colors represent LNM status, with red indicating positive cases and blue indicating negative cases. Four cases were negative but had high ZEB1 values (false-positive), twelve cases were negative with low ZEB1 values (true-negative), two cases were positive with low ZEB1 values (false-negative), and 23 cases were positive with high ZEB1 values (true-positive).

The Pearson correlation coefficient was significantly positive between ZEB1 expression and LNM, with a Pearson correlation coefficient of 0.68 (*p* = 4.98 × 10^−8^). Four of the 25 cases were node-negative, but they had high ZEB1 levels, whereas 12/25 cases were negative and had low ZEB1 levels. Two of the 25 cases were node-positive but had low ZEB1 levels, compared to 23/25 cases with high ZEB1 levels (Figure 5).

As shown in Table 3, there was a strong negative Pearson correlation coefficient of −0.59 (*p* = 36 × 10^−5^), which means that there was a relationship between the clinical stage and E-cadherin expression.

**Figure 6:**
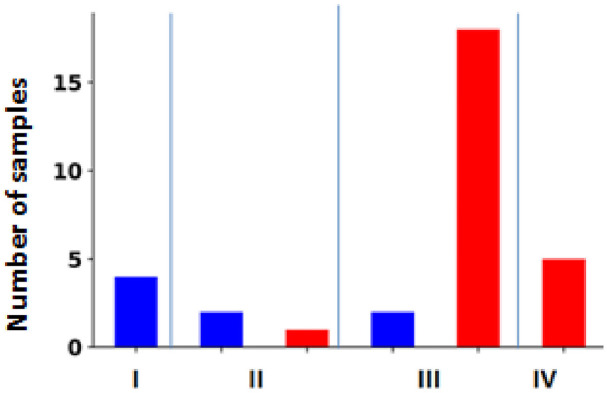
The correlation between the expression of ZEB1 and the stage of the tumor. This figure shows the expression of ZEB1 in different tumor clinical stages (I, II, III, and IV). The red bar represents a positive expression, and the blue bar represents a negative expression.

**Figure 7:**
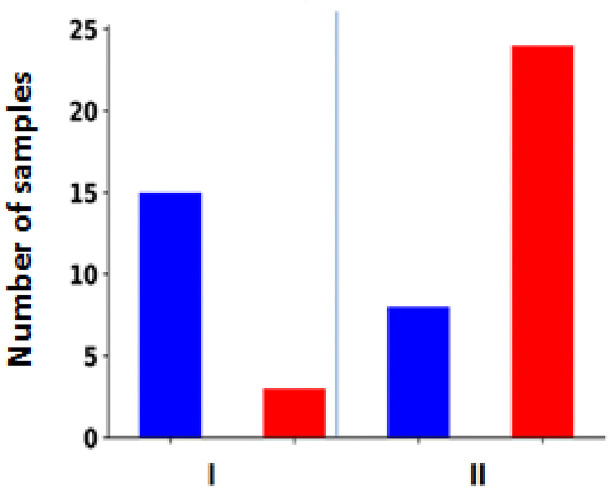
The correlation between ZEB1 and the tumor’s grade. This figure shows the expression of ZEB1 in different tumor grades (I and II). The red bar represents a positive expression, while the blue bar represents a.

Furthermore, there was a significant positive correlation between clinical stage and ZEB1 expression, as shown in Table 3, with a Pearson correlation coefficient of 0.72 (*p* = 2.62 × 10^−6^). Figure 6 shows the correlation between ZEB1 expression and cancer stage. All 4 cases (100%) in stage I showed negative immunostaining for ZEB1. Only 1/3 (33%) of stage II cases tested positive for ZEB1, whereas 18/20 (90%) of stage III cases tested positive for ZEB1 expression. In the stage IV group, all 5 cases (100%) showed positive immunostaining. For tumor grade, there was a negative correlation with E-cadherin expression, as shown in Table 3, with a Pearson correlation coefficient of −0.52 (*p* = 9.06 × 10^−5^). An immunohistochemical study of ZEB1 overexpression showed that ZEB1 was present in 15/18 cases (83%) of grade I and 24/32 cases (75% of grade II) (Figure 7). A significant positive correlation was observed for tumor grade, with a Pearson correlation coefficient of 0.56 (*p* = 2.18 × 10^−5^) between clinical grade and ZEB1 expression, as shown in Table 3.

**Figure 8:**
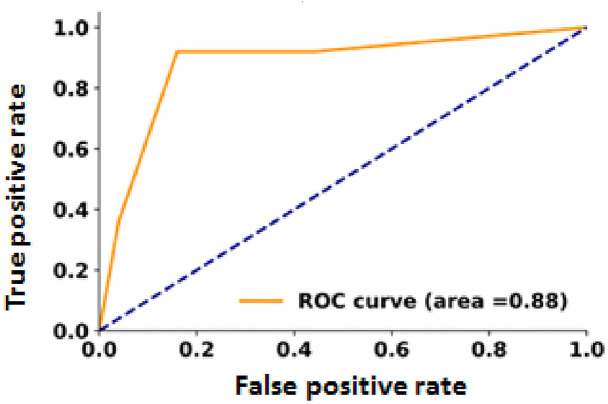
The model for the expression of ZEB1 in PTC patients is a receiver operator characteristic model. The area under the curve (AUC) = 0.88 (88%).

**Figure 9:**
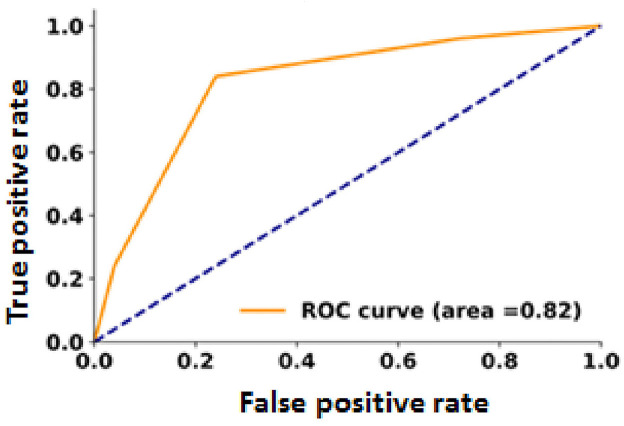
The model for the expression of E-cadherin in PTC patients is a receiver operator characteristic model. The area under the curve (AUC) = 0.82 (82%).

The receiver operating characteristic curves were plotted separately to determine the area under the curve (AUC) for each predictor and quantify the correlation for both ZEB1 and E-cadherin. The AUC for ZEB1 and E-cadherin was 0.88 and 0.82, respectively (Figures 8 and 9).

## DISCUSSION

This is a groundbreaking finding in this population as it is the first study to examine the prognostic significance of ZEB1 and E-cadherin biomarkers in Iraqi PTC patients. The incidence of PTC has increased dramatically worldwide over the past three decades despite stable mortality rates, in large part due to improvements in early detection and diagnostic methods worldwide.^10^ Approximately 50% of PTC patients have positive lymph node metastasis (LNM) at the time of surgery, as determined by lymphatic dissemination; Nodal involvement after surgery has been reported in 38.5%–88.8% of cases.^11^ Understanding the molecular mechanisms underlying the invasiveness of thyroid carcinoma is crucial because lymph nodes are the most common site of recurrence in thyroid cancer.^11^ Epithelial-mesenchymal transition (EMT), associated with advanced-stage carcinomas, is associated with the development of invasion and metastasis in cancer cells. This work focused on two important EMT biomarkers:^11^ E-cadherin and ZEB1.^12^

Strong associations were found between ZEB1 and E-cadherin levels and critical clinical variables such as LNM, tumor stage, and grade, indicating the potential utility of these biomarkers as prognostic indicators for PTC. According to our study, PTC was more common in younger women, with an average age of 38 years. While 80% of male patients had a higher incidence of thyroid cancer in nodules and the majority of patients had stage III LNM, 76% of female patients were lymph-node-negative, suggesting a better prognosis.^12^ In line with the findings of Machens et al., women typically have a better prognosis than men, most likely due to earlier diagnosis and lower disease stages.^13^

In terms of tumor grade, node-positive cases accounted for 92% of PTC patients, grade II tumors accounted for 64% of cases, and grade I tumors accounted for 36% of cases. E-cadherin is critical for cell-cell adhesion, influences epithelial cell morphogenesis, and is reduced during tumor invasion in a number of cancers.^14,15^ E-cadherin expression was significantly different between normal and malignant thyroid tissue according to our grading system (0–3), highlighting the tumor’s ability to invade and spread. In malignant thyroid tissues, the expression of ZEB1 was also significantly increased, especially in PTC with LNM, confirming its role in aggressive tumor behavior.^9,16,17^ Lower expression of E-cadherin in PTC cases supports the conclusions of Soares et al. and Brabant et al.,^18^ who found that PTC contained less E-cadherin than normal thyroid tissue.^15^ Furthermore, a strong inverse relationship was found between LNM and E-cadherin expression, with high E-cadherin expression observed in PTC without LNM.^19^ These results support those of Erdem et al.,^20^ who found an association between the presence of E-cadherin and the absence of metastasis and local invasion.^19^

E-cadherin expression peaked at 100% in stage I, declined dramatically to 30% in stage III, and finally reached 0% in stage IV. Reduced E-cadherin expression in distant metastases, local recurrences, and stage IV tumors suggests a role in later stages of tumor progression.^20^ In rare cases, tumors with local recurrences and distant metastases have E-cadherin positivity rates of more than 30%.^20^ A decrease in expression was noted with increasing tumor grade, with E-cadherin expression detected in 34% of grade II cases and 89% of grade I cases.^20^ Studies on meningiomas and bladder cancer have also shown that as tumor aggressiveness increases, E-cadherin expression decreases.^20,21^

ZEB1, a transcription factor involved in EMT, controls multiple malignant processes and functions as an oncogene in PTC cells.^9,16^ Previous studies have shown a strong correlation between stem cell characteristics and EMT, with ZEB1 playing a role in this phenotype.^17^ In this study, ZEB1 expression was either absent or decreased in the adjacent normal thyroid tissues but was positive in malignant PTC cells. This result is consistent with other studies, such as those by Liu et al., who discovered increased ZEB1 expression in lung adenocarcinoma tissues.^9^ Our study found that ZEB1-positive immunostaining was present in 54% of malignant cases, which is significantly higher than in normal tissues and suggests that overexpression of ZEB1 is a feature of thyroid cancer.

ZEB1 expression was highly correlated with LNM and was significantly higher in node-positive PTC cases than in node-negative cases. ZEB1 expression was significantly increased in PTC with LNM, whereas the majority of node-negative PTC cases exhibited negative staining, suggesting a possible function of ZEB1 in aggressive tumor behavior. This result is consistent with Chen et al. (2013), who proposed ZEB1 as a potential therapeutic target and risk factor for cervical cancer in pelvic LNM.^22^ ZEB1 expression was lowest at 33% in stage II and absent in stage I, with the highest percentages occurring in stages IV at 100% and in III at 90%. The remarkable association between ZEB1 and the clinical stage highlights its potential as a biomarker for the further development of PTC. ZEB1 positivity was detected in 83% of grade I cases and 75% of grade II cases; poorly differentiated tumors expressed ZEB1 more strongly than well-differentiated tumors.^22,23^

This study has a major impact on the prognosis and clinical treatment of PTC. Strong associations were found between ZEB1 and E-cadherin expression and key clinical features, suggesting that these biomarkers may be useful in identifying high-risk individuals and determining individualized treatment plans.^24^ To validate and extend these results, future investigations should focus on larger, multicenter studies with comprehensive clinical and molecular data.^25^ Furthermore, the consistency of these biomarkers in clinical practice would be improved by establishing uniform criteria for evaluating ZEB1 and E-cadherin expression in PTC, ultimately leading to better outcomes for patients.^26^

## CONCLUSION

Zinc finger E-box-binding homeobox 1 and E-cadherin are key biomarkers in PTC, strongly correlating with lymph node metastasis, tumor grade, and clinical stage. Their expression levels offer high predictive accuracy for identifying patients at risk of aggressive disease. Monitoring these markers can aid in early detection and personalized treatment. Future research should explore their broader clinical application to improve prognostic assessments and treatment outcomes for PTC patients.

## Acknowledgments

The authors thank all pathology lab staff at the teaching hospital in Najaf.

## Authors’ contribution

Dr. Israa granted final approval to the manuscript version and made significant contributions to the writing, conception, and planning of the study. Igbal oversaw the writing, data collection, analysis, and interpretation. Ali participated in the authorship and conducted a thorough assessment of the manuscript. Each author thoroughly reviewed and approved the final paper prior to its submission to the journal.

## Conflicts of interest

The authors do not have any conflicts of interest to disclose.

## Ethical declaration

This study (No. 14) has been approved by the Medical Ethics Committee of the Iraqi Ministry of Higher Education and Scientific Research.

## Informed consent

The study exempted the need for individual consent.

## Data availability

No additional data is available for this study.

## Figures and Tables

**Table:** 

**Value**	**Description**	**Cell staining**	**Interpretation**
0	No staining	0% of cells	Negative
1	Weak staining	1–20% of cells	Negative
2	Moderate staining	21–50% of cells	Positive
3	High staining	More than 50% of cells	Positive

**Table:** 

**Score**	**Description**	**Percentage of positive cells**	**Staining intensity**
0	Less than	25%	No staining
1	Light staining	26–50%	Light staining
2	Moderate staining	51–75%	Moderate staining
3	Strong staining	More than 75%	Strong staining

**Table 1.** Clinical and demographic characteristics of patients included in the immunohisto-chemical analysis.

**Category**	***n* (%)**
**Gender**	
Male	13 (26)
Female	37 (74)
**Age (years)**	
<50	39 (78)
50–60	8 (16)
>60	3 (6)
**Range**	18–92 years
**Median**	38 years
**Lymph node metastasis**	
With LNM	25 (50)
Without LNM	25 (50)
**Clinical stage**	
I	4 (8)
II	3 (6)
III	20 (40)
IV	5 (10)
Unknown	18 (36)
**Grade**	
I	18 (36)
II	32 (64)

Data are presented as *n* (%).

**Table 2.** Demographics of the 25 cases and 25 controls for E-cadherin and ZEB1 expression.

**Marker expression**	**Cases PTC with LNM (*n* = 25)**	**Control PTC without LNM (*n* = 25)**
**E-cadherin expression**		
Positive	6 (24%)	21 (84%)
Negative	19 (76%)	4 (16%)
**ZEB1 expression**		
Positive	23 (92%)	4 (16%)
Negative	2 (8%)	21 (84%)

LNM: lymph node metastases. Data are presented as *n* (%).

**Table 3.** Pearson correlation with the clinicopathological parameters.

**Parameter**	**E-cadherin**		**Pearson correlation coefficient (*r*) *p* value**	**ZEB1**		**Pearson correlation coefficient (*r*) *p* value**
	**+ *n*(%)**	**− *n* (%)**		**+ *n* (%)**	**− *n* (%)**	
**LNM**						
With LNM	6 (24%)	19 (76%)	−0.60 (3.76 × 0^−6^)	23 (92%)	2 (8%)	0.68 (4.98 × 10^−8^)
Without LNM	21 (84%)	4 (16%)		4 (16%)	21 (84%)	
**Total**	27 (54%)	23 (46%)		27 (54%)	23 (46%)	
**Stage**						
X	15 (83%)	3 (17%)	−0.59 (36 × 10^−5^)	3 (17%)	15 (83%)	0.72 (2.62 × 10^−6^)
I	4 (100%)	0		0	4 (100%)	
II	2 (67%)	1 (33%)		1 (33%)	2 (67%)	
III	6 (30%)	14 (70%)		18 (90%)	2 (10%)	
IV	0	5 (100%)		5 (100%)	0	
**Total**	27 (54%)	23 (46%)		27 (54%)	23 (46%)	
**Grade**						
I	16 (89%)	2 (11%)	−0.52 (9.06 × 10^−5^)	15 (83%)	3 (17%)	0.56 (2.18 × 10^−5^)
II	11 (34%)	21 (66%)		24 (75%)	8 (25%)	
**Total**	27 (54%)	23 (46%)		39 (78%)	11 (22%)	

Data are presented as *n* (%). *p* < 0.05, LNM: lymph node metastases.
